# Risk Factors of Female-Perpetrated Intimate Partner Violence among Hispanic Young Adults: Attachment Style, Emotional Dysregulation, and Negative Childhood Experiences

**DOI:** 10.3390/ijerph192113850

**Published:** 2022-10-25

**Authors:** Joahana Segundo, Arthur L. Cantos, Gabriela Ontiveros, K. Daniel O’Leary

**Affiliations:** 1 Department of Psychology, University of Houston, Houston, TX 77004, USA; 2 Department of Psychological Science, The University of Texas Rio Grande Valley, Edinburg, TX 78539, USA; 3 Department of Psychology, Stony Brook University, Stony Brook, NY 11794, USA

**Keywords:** intimate partner violence, emotion dysregulation, attachment style, adverse childhood experiences

## Abstract

This paper examined whether risk factors commonly associated with intimate partner violence (IPV) are associated with female-perpetrated physical IPV and female physical IPV victimization among young Hispanic women. It also examined how emotion dysregulation, impulsivity, and attachment style exacerbated these relationships. Furthermore, it investigates how these associations differ by the type of self-reported physical violence against their romantic partner. Based on the participants′ self-reported physical violence, they were classified into one of four groups: nonviolent, victim-only, perpetrator-only, and bidirectionally violent. Bidirectional violence was by far the most common form of violence reported. Utilizing self-report data from 360 young Hispanic women, we used binary logistic regression to examine potential predictors of physical IPV perpetration and victimization for each group. Results demonstrated that women in the bidirectionally violent group reported the highest levels of perpetration and victimization. Parental violence victimization, witnessing interparental violence, insecure attachment styles, and emotional dysregulation predicted physical IPV perpetration and victimization. These findings emphasize the need for effective interventions that include both members of the dyad and acknowledge the impact of women′s attachment style, emotion dysregulation, and adverse childhood experiences on female-perpetrated IPV and female IPV victimization.

## 1. Introduction

Intimate partner violence (IPV) is recognized as a serious public health issue in the United States (U.S.) that affects millions of people and results in short- and long-term physical and psychological consequences for victims [1,2]. IPV is composed of physical violence, sexual violence, psychological aggression, or stalking by a previous or current romantic partner [3]. In the U.S., approximately one in four women and one in ten men experience IPV by a partner and report being impacted by IPV [4]. Moreover, about 35% of female victims and 11% of male victims suffer physical injury as a result of IPV [4]. IPV is known to occur across all race-ethnicity groups in the U.S., and Hispanics are no exception [4,5]. In addition, physical aggression against a partner occurs in about 35% of young adult couples [6,7]. Every year, approximately 80% of dating college students will perpetrate or be victimized by psychological aggression, and 20–30% will perpetrate or be victimized by physical aggression in their dating relationships [8,9].

Despite women perpetrating IPV at similar or higher rates than men [10,11], in representative or community samples, research on female-perpetrated IPV among Hispanic couples is less frequently investigated. Understanding the risk factors associated with female perpetrated violence among young Hispanic women is essential for developing practical clinical interventions for this underrepresented group. The existing IPV literature primarily focuses on non-Hispanic Whites despite the growing number of Hispanics residing in the U.S. [12]. In addition, Hispanic couples have a higher risk of engaging in IPV than White couples [13]. More specifically, the rates of man-to-woman violence and woman-to-man violence are higher among Hispanic couples (17% and 21%) than in White couples (12% and 16%) [13]. However, IPV prevalence differences between Hispanics and Whites in crude rates are reduced after controlling for socioeconomic circumstances and alcohol use [14]. Additionally, it is important to consider intra-ethnic considerations because of the dramatic differences in IPV among Hispanics from different countries of origin [15].

Although research has demonstrated that most IPV is bidirectional [16,17], IPV interventions primarily focus on men′s violence toward women [18,19]. The lack of clinical interventions for female perpetrators of IPV may be due to a limited scientific understanding of the factors that influence IPV perpetration among this group. The relationship between IPV perpetration and gender is an aspect of IPV that is still not well understood due to the lack of research on this topic. As emphasized by Jose & O′Leary [20], a key problem in this research literature is the failure to differentiate between clinical and representative samples. In classic research by Straus and Gelles [5], with large representative community samples in the U.S., men and women perpetrated physical aggression against their partners at about equal rates, with both at approximately 12%. In samples of men and women seeking relationship or marital therapy, the perpetration of physical aggression against a partner across three different states was between 50–60%, and there were no significant differences between perpetration rates of men and women [20]. However, even in representative community samples in which there is physical aggression, women are more fearful of men than vice versa [21]. Further, it is almost impossible to know the rates of perpetration of physical aggression against partners in the most severe cases of partner violence. Still, it is evident at the very most end of the aggression continuum that males predominate. For instance, in a study using partner homicide data from the Federal Bureau of Investigation (FBI) [22], results demonstrated that 68% of the homicide victims were women.

At ages when partner aggression is just beginning to occur, such as in junior and senior high school years, it appears that prevalence rates of physical aggression against a partner are somewhat higher for female students than male students [23,24]. Furthermore, some research on college students shows that women perpetrate as much, if not more, psychological and physical aggression than men [25,26] and are more likely to initiate aggression [27]. According to Monson and Langhinrichsen-Rohling [28], the typical female IPV perpetrator engages in low levels of bidirectional IPV [29] and may do so due to poor intimacy management in their romantic relationship.

Different theories of interpersonal violence have been used to explain IPV and adult attachment theory [30] being one of them. Attachment problems are consistent risk factors for IPV [31,32,33]. According to Bowlby [34], attachment is a behavioral system that organizes human behaviors to increase survival and adaptation. The attachment system is activated when a person is confronted with a situation that risks their survival. Once activated, its first response is to seek proximity to an attachment figure (i.e., romantic partner and parents) [34]. A secure attachment, as well as the feeling of being deserving of others′ love (i.e., working models of self) and confidence that others will provide support in times of need (i.e., working models of others), is developed if attachment figures respond consistently to the individual′s attachment needs [35]. These mental representations of self and others can be described in two dimensions: anxious attachment (i.e., anxiety over abandonment) and avoidance attachment (i.e., avoidance of intimacy) [36].

Relationship researchers view IPV as a dysfunctional attempt to remain close to a partner when threats to attachment needs are perceived [37]. Perceived threats to attachment needs by insecure partners may generate differences in perspectives, ultimately resulting in perpetration or victimization of violence or a combination of both. For instance, anxious attachment is a consistent predictor of both perpetration and victimization of IPV in both men and women [38,39]. Attachment-related anxiety is associated with intimate partner aggression among dating and married couples [39,40]. Such relationships are mixed for attachment avoidance. In particular, Cummings-Robeau et al. [41] found an association between attachment-related avoidance and IPV. In contrast, Miga et al. [42] did not find this attachment style related to such violence. However, other factors, such as interactions of different attachment styles may be at play, as illustrated by research that showed that attachment anxiety, impulsivity, and an interaction effect between attachment avoidance and partner′s attachment anxiety were associated with self-reported, but not partner-reported, male perpetration [43].

There is also abundant evidence that suggests that emotion dysregulation is a predictor of IPV perpetration. It is theorized that difficulties in emotion regulation are due to a biological predisposition to react emotionally and to having been raised in an invalidating environment [44]. Over time, difficulties in emotion regulation may manifest in behavioral problems and disorders associated with IPV perpetration, such as antisocial personality disorder [45,46], borderline personality disorder [46,47,48], and depression [49,50]. Various studies have found emotion regulation difficulties to be positively associated with IPV perpetration [51,52], including studies with young adults in romantic relationships [43,53,54]. Due to the substantial evidence demonstrating the importance of the role of emotion regulation in IPV perpetration, it is important to investigate how it may affect female perpetrators.

One′s upbringing or negative childhood experiences may also affect one′s likelihood of perpetrating aggression. Individuals′ background and traumatic childhood experiences, specifically witnessing interparental violence and experiencing childhood physical abuse, have been consistently found to be precursors for IPV perpetration and victimization [55,56,57] and recidivism [58]. Some evidence suggests an increased risk of perpetration for individuals reporting childhood abuse or witnessing interparental violence [59,60]. In addition, exposure to IPV and parental violence victimization may also lead to an increased risk of victimization of IPV [61,62,63]. However, the mechanisms of this relationship need to be further investigated.

The research herein investigated the patterns of physical IPV perpetration and victimization among young adult Hispanic women. Specifically, we examined whether the violence is predominantly bidirectional, as in the previously cited studies, or unilateral, and the frequency of physical aggression in the relationship reported as IPV perpetration or IPV victimization. Lastly, we examined how emotion dysregulation, adult attachment style, impulsivity, and prior exposure to violence in childhood may exacerbate female physical IPV perpetration and victimization. In this context, the hypotheses developed in line with the general purpose of the current research are as follows:

**Hypothesis** **1** **(H1):** Bidirectional violence will be the most frequent pattern of IPV for all groups and participants in the bidirectionally violent group will have the highest frequency of IPV (perpetration and victimization) in their relationship.

**Hypothesis** **2** **(H2):** The bidirectionally violent group and the perpetrator-only group will report higher levels of anxious attachment and emotional regulation difficulties than the nonviolent group and the victim-only group.

**Hypothesis** **3** **(H3):** Parental violence victimization and witnessing interparental violence will be positively associated with IPV perpetration and victimization.

**Hypothesis** **4** **(H4):** Insecure attachment (anxious and avoidant) will moderate the association between parental violence victimization and IPV perpetration and IPV victimization.

**Hypothesis** **5** **(H5):** Insecure attachment (anxious and avoidant) will moderate the association between witnessing interparental violence and IPV perpetration and victimization.

**Hypothesis** **6** **(H6):** Difficulties with emotion regulation and impulsivity will moderate the association between anxious attachment style and IPV perpetration and victimization.

**Hypothesis** **7** **(H7)** : Difficulties with emotion regulation and impulsivity will moderate the association between avoidant attachment style and IPV perpetration and victimization.

**Hypothesis** **8** **(H8):** Difficulties with emotion regulation and impulsivity will moderate the association between parental violence victimization and IPV perpetration.

**Hypothesis** **9** **(H9):** Difficulties with emotion regulation and impulsivity will moderate the association between witnessing interparental violence and IPV perpetration and victimization.

## 2. Materials and Methods

### 2.1. Participants

Participants were 360 women undergraduate students from a southern Hispanic-Serving Institution. Research credit was granted to participants for a psychology course. To be eligible for the study, participants were required to be currently or previously involved in a romantic relationship of at least one month and be 18 years or older to participate. The exclusion criteria were failure to meet the inclusion criteria or refusal to give informed consent.

### 2.2. Procedure

Participants accessed a web link to the online survey via Qualtrics, which first displayed the cover letter informing participants about the study and requested their consent to participate in this research. After providing consent, participants were asked to complete demographic items, an item on relationship status, items about the relationship, an item on general violence, items on witnessing interparental violence, and scales assessing IPV (perpetration and victimization), adult romantic attachment, and emotional regulation. Then, participants were presented with information about how to receive credit for their participation.

### 2.3. Measures

#### 2.3.1. Relationship and Demographic Items

Participants completed demographic items that included age, self and partner′s gender, and ethnicity/race. The following items regarding participants′ current/latest relationship were also included: relationship status, relationship length, cohabitation status, and cohabitation length.

#### 2.3.2. Witnessing Interparental Violence

The following items were included to assess experiences with witnessing interparental violence: (1) “During childhood, how often did your parents hit you?”, (2) “I witnessed my father hitting my mother. If this statement is true, how often did you witness this event?” (3) “I witnessed my mother hitting my father. If this statement is true, how often did you witness this event?” and (4) “I witnessed my father and mother hit each other. If this statement is true, how often did you witness this event?” A 5-point Likert scale was used with choices ranging from 1 (*never*) to 5 (*very frequently*). A higher score indicates a higher frequency of witnessing interparental violence. Internal consistency for witnessing interparental violence was α = 0.92.

#### 2.3.3. Intimate Partner Violence

Physical IPV was assessed using the Revised Conflicts Tactics Scale (CTS-2) [64]. This measure demonstrates good internal consistency ranging from 0.79 to 0.95. Using this scale of 78 items, participants reported whether they were involved in acts of relationship violence, such as perpetration or victimization, with an intimate partner in the past year. The items are organized on an 8-point Likert-type scale (1 = *Once in the past year*; 2 = *Twice in the past year*; 3 = *3 to 5 times in the past year*; 4 = *6 to 10 times in the past year*; 5 = *11 to 20 times in the past year*; 6 = *More than 20 times in the past year*; 7 = *Not in the past year, but it did happen before*; 0 = *This has never happened*). Based on recommendations by Straus et al. [64], scores of 7 are then converted to 0 to demonstrate that the behavior did not occur in the past year; scores of 3 are converted to the midrange value of 4; scores of 4 are converted to midrange value of 8; scores of 5 are converted to the midrange value of 15, and scores of 6 are converted to the maximum value of 25. Then, the CTS-2 items are summed across the following four subscales: psychological aggression, physical assault, sexual coercion, and injury (only the physical assault scale is reported in this study). Within each subscale, the items are characterized into minor and severe behaviors (based on scoring methods). Participants are then classified into one of four IPV history groups based on their responses to the 12 items on the physical assault subscale. Internal consistency for the present study was α = 0.93 for the physical assault scale.

#### 2.3.4. Attachment Dimensions

Adult romantic attachment style was measured using the Experiences in Close Relationships Revised questionnaire (ECR-R) [36,65]. The ECR-R is a 36-item self-report attachment measure with excellent internal consistency reliability of 0.90 or above. This measure provides scores on two subscales assessing different theoretical dimensions of attachment style: attachment avoidance (i.e., discomfort with closeness and depending on others) and attachment anxiety (i.e., fear of rejection and abandonment). Participants indicated how strongly they agreed with each item using a Likert-type scale (1 = *Strongly agree* to 7 = *Strongly disagree*). The ECR-R is scored by computing a mean value for each subscale after reverse coding the appropriate items. Internal consistency for attachment avoidance was α = 0.90, and internal consistency for attachment anxiety was α = 0.91 in the present study.

#### 2.3.5. Emotion Regulation

Emotion regulation was measured using the brief version of the Difficulties in Emotion Regulation Scale (DERS-18) [66]. The DERS-18 is an 18-item self-report questionnaire with high internal consistency reliability ranging from 0.70 to 0.90, designed to assess multiple aspects of emotion regulation. It is composed of six subscales: awareness, clarity, goals, impulse, nonacceptance, and strategies. The items are organized on a 5-point Likert-type scale [1 = *Almost never (0–10%)*; 2 = *Sometimes (11–35%)*; 3 = *About half the time (36–65%)*; 4 = *Most of the time (66–90%)*; and 5 = *Almost always (91–100%)*]. After items 1, 4, and 6 are reverse-coded and the remaining items are scored, items′ scores will be summed to obtain a total score. Higher scores suggest greater problems with emotion regulation. This study used a total score based on all six subscales. The present study′s internal consistencies for the DERS-18 and its impulse subscale were α = 0.86 and α = 0.88, respectively.

### 2.4. Statistical Analysis Strategy

The analyses proceeded in multiple steps. First, study participants were classified into one of the following mutually exclusive categories based on self-reported physical IPV perpetration and/or victimization: no-violence, which included participants who endorsed no forms of perpetration or victimization, perpetrator-only, which included participants who endorsed at least one form of perpetration and no forms of victimization, victim-only which included participants who endorsed at least one form of victimization and no forms of perpetration, and bidirectionally violent which included participants who endorsed at least one form of victimization and perpetration. Second, descriptive statistics were conducted to examine demographic variables and the prevalence of physical IPV in each of the four categories. Third, chi-square tests of independence were used to assess for statistically significant differences in the prevalence of study variables (e.g., anxious attachment, impulsivity) within each category. Fourth, bivariate correlation analyses were conducted to evaluate the association between continuous demographic variables (e.g., length of cohabitation with their partner), study variables (e.g., frequency of parental physical victimization), and IPV perpetration and victimization. Fifth, a series of models were created from binary logistic regression analyses to examine potential predictors associated with physical IPV perpetration and victimization.

All statistical analyses were conducted using IBM Statistical Package for the Social Sciences (SPSS) version 26 (International Business Machines Corporations, Armonk, NY, USA). Missing data were assumed to be missing completely at random.

## 3. Results

### 3.1. Descriptive Statistics and Classification of Physical IPV Groups

In our study, we included 360 women who indicated having been in a romantic relationship of at least one month in the last 12 months or are currently in a romantic relationship of at least one month. Demographic characteristics of the study sample are presented in Table 1.

In addition, participants were classified into one of four physical IPV groups (i.e., nonviolent, victim-only, perpetrator-only, bidirectionally violent) based on their self-reports of physical violence on the CTS-2 (see Table 1). Specifically, if participants reported no violence in their relationship, they would be classified as nonviolent. If participants reported being a victim of physical assault in their relationship and no perpetration of violence, they were classified as victim-only. If participants reported perpetrating physical violence toward their partner and no victimization of IPV in their relationship, they were classified as perpetrator-only. Lastly, if participants reported both victimization and perpetration of physical violence in their relationship, they were classified as bidirectionally violent. The means for the observed risk factors for the overall sample and IPV groups are presented in Table 2.

### 3.2. Hypotheses

#### 3.2.1. Hypothesis 1

The first hypothesis was that participants in the bidirectionally violent category, including those who endorsed at least one form of victimization and perpetration, would have a higher frequency of physical IPV perpetration and victimization in their relationship when compared to the perpetrator-only and victim-only categories. Table 3 provides the frequency with which females endorsed physical violence perpetration and/or victimization. Among the total sample (*n* = 360), 242 (67.2%) reported no perpetration, 64 (17.8%) reported bidirectional violence, 39 (10.8%) reported being a perpetrator only, and 15 (4.2%) reported being a victim only. Thus, of those reporting violence (32.8%), there was a higher frequency of bidirectional violence (54.2%) as compared to only perpetration and only victimization (33.0% and 12.7%, respectively).

Further, a one-way ANOVA was used to compare total physical IPV perpetration (as reported in the CTS2) in the past year by members for the bidirectionally violent, perpetrator-only, and victim-only categories. Physical IPV perpetration differed significantly across the three groups, F(3, 356) = 25.27, *p* < 0.001. Tukey’s HSD post hoc comparisons of the groups indicated that participants in the category of bidirectionally violent category reported significantly higher levels of physical IPV perpetration when compared to both the perpetrator-only (M= 19.74, 95% CI [11.59, 27.89]) and victim-only (M= 22.28, 95% CI [10.77, 33.79]) categories. These results suggest that participants in the bidirectionally violent category reported more physical IPV perpetration than any other category.

Additionally, a one-way ANOVA was used to compare total physical IPV victimization (as reported in the CTS2) in the past year by members of the bidirectionally violent, perpetrator-only, and victim-only categories. Physical IPV perpetration differed significantly across the three groups, F(3, 355) = 37.20, *p* < 0.001. Tukey’s HSD post hoc comparisons of the groups indicated that participants in the bidirectionally violent category reported significantly higher levels of physical IPV victimization when compared to both the perpetrator only (M= 20.09, 95% CI [12.89, 27.30]) and victim only (M= 18.09, 95% CI [7.92, 28.27]) categories. These results indicate that participants in the bidirectionally violent category reported more physical IPV victimization than any other category.

#### 3.2.2. Hypothesis 2

The second hypothesis was that participants would report higher levels of anxious attachment, avoidant attachment, impulsivity, and emotion regulation difficulties if they were in the bidirectionally violent or perpetrator-only categories compared to the victim-only and nonviolent categories. Thus, one-way ANOVA analyses were used to investigate if these risk factors have individual, significant effects on physical IPV perpetration and victimization in each category.

Anxious attachment differed significantly across the four groups, F(3, 356) = 3.14, *p* = 0.025. Tukey’s post hoc comparisons of the groups indicated that participants in the bidirectionally violent category (M= 9.24, 95% CI [0.99, 17.48]) reported significantly higher levels of anxious attachment when compared to the nonviolent category. Comparisons between the bidirectionally violent category and the perpetrator-only and victim-only categories were not statistically significant at *p* < 0.05.

Avoidant attachment differed significantly across the four groups, F(3, 356) = 6.49, *p* < 0.001. Tukey’s post hoc comparisons of the groups indicated that participants in the bidirectionally violent category reported significantly higher levels of avoidant attachment when compared to the nonviolent category (M= 11.94, 95% CI [4.58, 19.29]) and perpetrator only (M= 14.23, 95% CI [3.59, 24.86]) category. Comparisons between the bidirectionally violent and victim-only categories were not statistically significant at *p* < 0.05.

Impulsivity differed significantly across the four groups, F(3, 345) = 3.49, *p* = 0.016. Tukey’s post hoc comparisons of the groups indicated that participants in the bidirectionally violent category (M= 2.25, 95% CI [0.37, 4.12]) reported significantly higher levels of impulsivity when compared to the nonviolent category. Comparisons between the bidirectionally violent category and the perpetrator-only and victim-only categories were not statistically significant at *p* < 0.05.

Emotion regulation difficulties (as reported by the total DERS score) differed significantly across the four groups, F(3, 346) = 2.68, *p* = 0.047. Tukey’s post hoc comparisons of the groups indicated that participants in the category of bidirectionally violent (M = 8.97, 95% CI [−0.35, 18.29]) reported significantly higher levels of emotion regulation when compared to the nonviolent category. Comparisons between the bidirectionally violent category and the perpetrator-only and victim-only categories were not statistically significant at *p* < 0.05.

#### 3.2.3. Hypothesis 3

The third hypothesis was that there would be positive associations between parental violence victimization and witnessing interparental violence and physical IPV perpetration and victimization. There was a significant positive association between physical IPV perpetration and frequency of parental physical victimization (r = 0.16, *p* < 0.01; Table 4), witnessing both parents hit each other (r = 0.12, *p* < 0.05), and witnessing one’s father hit one’s mother (r = 0.11, *p* < 0.01). In addition, there was a significant and positive association between physical IPV victimization and frequency of parental physical victimization (r = 0.22, *p* < 0.01) and witnessing one’s mother hit one’s father (r = 0.12, *p* < 0.05).

#### 3.2.4. Hypothesis 4

The fourth hypothesis was that insecure attachment (anxious and avoidant) will moderate the relationship between parental violence victimization and physical IPV perpetration and victimization (irrespective of each other). Because the bivariate correlation between attachment anxiety and avoidant attachment was high (r = 0.51, *p* < 0.01), there would likely be shared variance between them that could explain physical IPV perpetration and victimization. Thus, separate regressions were conducted to examine the associations between perpetration, victimization, parental violence victimization, and the two attachment dimensions.

Results from the binary logistic regression assessing the relationship between parental physical victimization, anxious attachment and physical IPV perpetration indicated that parental physical victimization was a significant predictor of physical IPV perpetration (B = 0.266, *p* = 0.004). The full model explained between 3.9% (Cox & Snell R2) and 5.6% (Nagelkerke R2) of the variance. Anxious attachment was not a significant predictor of physical IPV perpetration (*p* > 0.05). When the moderation of anxious attachment on the association between parental physical victimization and physical IPV perpetration was assessed, results indicated that there were no significant interactions (*p* > 0.05), thus there is no moderation effect of anxious attachment on the relationship between parental physical victimization and IPV perpetration.

Results from the binary logistic regression assessing the relationship between parental physical victimization, avoidant attachment and physical IPV perpetration indicated that parental physical victimization and avoidant attachment were significant predictors of physical IPV perpetration (B = 0.244, *p* = 0.009; B = 0.012, *p* = 0.035, respectively). The full model explained between 3.8% (Cox & Snell R2) and 5.5% (Nagelkerke R2) of the variance. When the moderation of avoidant attachment on the relationship between parental physical victimization and physical IPV perpetration was analyzed, the results indicated no significant interactions (*p* > 0.05).

Again, a binary logistic regression was used to assess the relationship between parental physical victimization, anxious attachment, and physical IPV victimization. Both parental physical victimization and anxious attachment were significant predictors of physical IPV victimization (B = 0.390, *p* < 0.001; B = 0.013, *p* = 0.021, respectively). The full model explained between 6.5% (Cox & Snell R2) and 9.9% (Nagelkerke R2) of the variance. When the moderation of anxious attachment on the association between parental physical victimization and physical IPV victimization was assessed, the results indicated no significant interaction (*p* > 0.05).

The relationship between parental physical victimization, avoidant attachment, and physical IPV victimization was assessed via binary logistic regression. Both parental physical victimization and avoidant attachment were significant predictors of physical IPV victimization (B = 0.410, *p* < 0.001; B = 0.021, *p* < 0.001, respectively). The full model explained between 7.7% (Cox & Snell R2) and 11.8% (Nagelkerke R2) of the variance. When the moderation of avoidant attachment on the relationship between parental physical victimization and physical IPV victimization was assessed, the results indicated no significant interaction (*p* > 0.05).

#### 3.2.5. Hypothesis 5

The fifth hypothesis was that insecure attachment (anxious and avoidant) would moderate the relationship between witnessing interparental violence and physical IPV perpetration and victimization. Again, separate regressions were conducted to examine the associations between perpetration, victimization, witnessing interparental violence, and the two attachment dimensions.

Results from the binary logistic regression assessing the relationship between witnessing interparental violence, anxious attachment and physical IPV perpetration indicated that witnessing interparental violence was a significant predictor of physical IPV perpetration (B = 0.167 *p* = 0.016). The full model explained between 2.7% (Cox & Snell R2) and 3.8% (Nagelkerke R2) of the variance. Anxious attachment was not a significant predictor of physical IPV perpetration (*p* > 0.05). When the moderation of anxious attachment on the association between witnessing interparental violence and physical IPV perpetration was assessed, results indicated that there were no significant interactions (*p* > 0.05). Thus there is no moderation effect of anxious attachment on the relationship between witnessing interparental violence and IPV perpetration.

Results from the binary logistic regression assessing the relationship between witnessing interparental violence, avoidant attachment and physical IPV perpetration indicated that witnessing interparental violence and avoidant attachment were significant predictors of physical IPV perpetration (B = 0.167, *p* = 0.016; B = 0.014, *p* = 0.011, respectively). The full model explained between 3.4% (Cox & Snell R2) and 4.9% (Nagelkerke R2) of the variance. When the moderation of avoidant attachment on the relationship between witnessing interparental violence and physical IPV perpetration was analyzed, the results indicated no significant interactions (*p* > 0.05).

Again, a binary logistic regression was used to assess the relationship between witnessing interparental violence, anxious attachment, and physical IPV victimization. Both witnessing interparental violence and anxious attachment were significant predictors of physical IPV victimization (B = 0.166, *p* = 0.018; B = 0.014, *p* = 0.014, respectively). The full model explained between 3.4% (Cox & Snell R2) and 5.2% (Nagelkerke R2) of the variance. When the moderation of anxious attachment on the association between witnessing interparental violence and physical IPV victimization was assessed, the results indicated no significant interaction (*p* > 0.05).

The relationship between witnessing interparental violence, avoidant attachment and physical IPV victimization was assessed via binary logistic regression. Both witnessing interparental violence and avoidant attachment were significant predictors of physical IPV victimization (B = 0.166, *p* = 0.018; B = 0.024, *p* < 0.001, respectively). The full model explained between 5.7% (Cox & Snell R2) and 8.7% (Nagelkerke R2) of the variance. When the moderation of avoidant attachment on the relationship between witnessing interparental violence and physical IPV victimization was assessed, the results indicated no significant interaction (*p* > 0.05).

#### 3.2.6. Hypothesis 6

The sixth hypothesis was that emotion regulation difficulties and impulsivity will moderate the relationship between anxious attachment style and IPV perpetration and victimization. Emotion regulation was assessed using the total DERS score and separately using only impulsivity, a subscale of the total score.

Results from the binary logistic regression assessing the relationship between emotion regulation, anxious attachment and physical IPV perpetration indicated that emotion regulation and anxious attachment were significant predictors of physical IPV perpetration (B = 0.029, *p* = 0.031; B = 0.047, *p* = 0.016, respectively). The full model explained between 2.5% (Cox & Snell R2) and 3.6% (Nagelkerke R2) of the variance. When the moderation of emotion regulation on the relationship between anxious attachment and physical IPV perpetration was assessed, the results indicated a significant interaction (B = 0.001, *p* = 0.034).

When assessing the relationship between impulsivity, anxious attachment and physical IPV perpetration, results indicated that anxious attachment was a significant predictor of physical IPV perpetration (B = 0.011, *p* = 0.038). The full model explained between 2.5% (Cox & Snell R2) and 3.5% (Nagelkerke R2) of the variance. When the moderation of impulsivity on the relationship between anxious attachment and physical IPV perpetration was assessed, the results indicated no significant interaction (*p* > 0.05).

When the same analysis was conducted to assess the moderating effects of emotion regulation on the relationship between anxious attachment and physical IPV victimization, emotion regulation and anxious attachment were significant predictors of physical IPV victimization (B = 0.047, *p* = 0.003; B = 0.062, *p* = 0.008, respectively). The full model explained between 4.7% (Cox & Snell R2) and 7.2% (Nagelkerke R2) of the variance. When the moderation of emotion regulation on the relationship between anxious attachment and physical IPV victimization was assessed, the results indicated a significant interaction (B = −0.001, *p* = 0.015).

When assessing the relationship between impulsivity, anxious attachment and physical IPV victimization, results indicated that impulsivity and anxious attachment were significant predictors of physical IPV victimization (B = 0.211, *p* = 0.004; B = 0.043, *p* = 0.010, respectively). The full model explained between 4.7% (Cox & Snell R2) and 7.3% (Nagelkerke R2) of the variance. When the moderation of impulsivity on the relationship between anxious attachment and physical IPV victimization was assessed, the results indicated a significant interaction (B = −0.002, *p* = 0.026).

#### 3.2.7. Hypothesis 7

The seventh hypothesis was that emotion regulation difficulties and impulsivity will moderate the relationship between avoidant attachment style and IPV perpetration and victimization. Emotion regulation was assessed using the total DERS score and separately using only impulsivity, a subscale of the total score.

Results from the binary logistic regression assessing the relationship between emotion regulation, avoidant attachment and physical IPV perpetration indicated that emotion regulation and avoidant attachment were significant predictors of physical IPV perpetration (B = 0.029, *p* = 0.029; B = 0.064, *p* = 0.009, respectively). The full model explained between 3.5% (Cox & Snell R2) and 5.0% (Nagelkerke R2) of the variance. When the moderation of emotion regulation on the relationship between avoidant attachment and physical IPV perpetration was assessed, the results indicated a significant interaction (B = −0.001, *p* = 0.034).

When assessing the relationship between impulsivity, avoidant attachment, and physical IPV perpetration, results indicated that avoidant attachment was a significant predictor of physical IPV perpetration (B = 0.035, *p* = 0.042). The full model explained between 3.6% (Cox & Snell R2) and 5.1% (Nagelkerke R2) of the variance. When the moderation of impulsivity on the relationship between avoidant attachment and physical IPV perpetration was assessed, the results indicated no significant interaction (*p* > 0.05).

When the same analysis was conducted to assess the moderating effects of emotion regulation on the relationship between avoidant attachment and physical IPV victimization, emotion regulation and avoidant attachment were significant predictors of physical IPV victimization (B = 0.015, *p* = 0.003; B = 0.020, *p* = 0.003, respectively). The full model explained between 5.1% (Cox & Snell R2) and 7.9% (Nagelkerke R2) of the variance. When the moderation of emotion regulation on the relationship between avoidant attachment and physical IPV victimization was assessed, the results indicated no significant interaction (*p* > 0.05).

When assessing the relationship between impulsivity, avoidant attachment and physical IPV victimization, results indicated that impulsivity and avoidant attachment were significant predictors of physical IPV victimization (B = 0.057, *p* = 0.030; B = 0.021, *p* = 0.001, respectively). The full model explained between 5.7% (Cox & Snell R2) and 8.7% (Nagelkerke R2) of the variance. When the moderation of impulsivity on the relationship between avoidant attachment and physical IPV victimization was assessed, the results indicated no significant interaction (*p* > 0.05).

#### 3.2.8. Hypothesis 8

Hypothesis 8 was that difficulties with emotion regulation and impulsivity will moderate the association between parental violence victimization and IPV perpetration. Emotion regulation was assessed using the total DERS score and separately using only impulsivity, a subscale of the total score.

Results from the binary logistic regression assessing the relationship between emotion regulation, parental violence victimization and physical IPV perpetration indicated that parental violence victimization was a significant predictor of physical IPV perpetration (B = 0.248, *p* = 0.009). The full model explained between 2.8% (Cox & Snell R2) and 4.1% (Nagelkerke R2) of the variance. When the moderation of emotion regulation on the relationship between parental violence victimization and physical IPV perpetration was assessed, the interaction was not significant (*p* > 0.05).

When assessing the relationship between impulsivity, parental violence victimization, and physical IPV perpetration, results indicated that parental violence victimization and impulsivity were significant predictors of physical IPV perpetration (B = 0.292, *p* = 0.002; B = 0.049, *p* = 0.035, respectively). The full model explained between 4.5% (Cox & Snell R2) and 6.5% (Nagelkerke R2) of the variance. When the moderation of impulsivity on the relationship between parental violence victimization and physical IPV perpetration was assessed, the results indicated no significant interaction (*p* > 0.05).

#### 3.2.9. Hypothesis 9

The ninth hypothesis is that difficulties with emotion regulation and impulsivity will moderate the association between witnessing interparental violence and IPV perpetration and victimization.

Emotion regulation was assessed using the total DERS score and separately using only impulsivity, a subscale of the total score. Results from the binary logistic regression assessing the relationship between emotion regulation, witnessing interparental violence, and physical IPV perpetration indicated that witnessing interparental violence was a significant predictor of physical IPV perpetration (B = 0.139, *p* = 0.048). The full model explained between 1.9% (Cox & Snell R2) and 2.8% (Nagelkerke R2) of the variance. When the moderation of emotion regulation on the relationship between witnessing interparental violence and physical IPV perpetration was assessed, the interaction was not significant (*p* > 0.05).

Results from the binary logistic regression assessing the relationship between impulsivity, witnessing interparental violence, and physical IPV perpetration indicated that impulsivity and witnessing interparental violence were significant predictors of physical IPV victimization (B = 0.049, *p* = 0.035; B = 0.159, *p* = 0.022, respectively). The full model explained between 2.8% (Cox & Snell R2) and 4.0% (Nagelkerke R2) of the variance. When the moderation of impulsivity on the relationship between witnessing interparental violence and physical IPV perpetration was assessed, the interaction was not significant (*p* > 0.05).

When the same analysis was conducted to assess the moderating effects of emotion regulation on the relationship between witnessing interparental violence and physical IPV victimization, emotion regulation and witnessing interparental violence were significant predictors of physical IPV victimization (B = 0.014, *p* = 0.007; B = 0.144, *p* = 0.046, respectively). The full model explained between 3.7% (Cox & Snell R2) and 5.7% (Nagelkerke R2) of the variance. When the moderation of emotion regulation on the relationship between witnessing interparental violence and physical IPV victimization was assessed, results indicated that the interaction was not significant (*p* > 0.05).

When the moderating effects of impulsivity on the relationship between witnessing interparental violence and physical IPV victimization was assessed, impulsivity and witnessing interparental violence were significant predictors of physical IPV victimization (B = 0.068, *p* = 0.007; B = 0.151, *p* = 0.040, respectively). The full model explained between 3.8% (Cox & Snell R2) and 5.9% (Nagelkerke R2) of the variance. When the moderation of impulsivity on the relationship between witnessing interparental violence and physical IPV victimization was assessed, results indicated that the interaction was not significant (*p* > 0.05).

## 4. Discussion

In a sample of young adult Hispanic females, 32% reported a history of violence in their relationships with a significant other. Of these, the majority, 54.2% reported both perpetrating and experiencing violence (bidirectional), 33% reported perpetrating violence only (perpetrator-only), and only 12.7% reported being a victim only. As predicted, women who reported bidirectional violence also reported perpetrating more physical violence and being more physically victimized than both the women who reported only perpetrating violence and the women who reported having been victimized only. Women who both perpetrated and were victimized reported higher levels of anxious attachment than women who did not report any violence but did not differ from the women who only perpetrated violence and those who were only victimized. Women who reported bidirectional violence also reported higher levels of avoidant attachment than the women who reported no violence and the women who reported only perpetrating violence but did not differ from the women who reported being victims only. Similarly, women who reported both experiencing and perpetrating violence reported more emotional regulation difficulties in general and more impulsivity than women who did not report violence but they did not differ from the women who reported only perpetrating violence and those who reported only being victimized did not differ from those women who reported no violence.

In this sample of Hispanic young adult females, there was a significant positive association between physical IPV perpetration and avoidant attachment, frequency of parental physical victimization, witnessing both parents hit each other, to have seen their father hit mother, impulse control difficulties, difficulty engaging in goal-directed behavior, nonacceptance of emotional responses, and limited access to emotion regulation strategies. In addition, there was a significant and positive association between physical IPV victimization and anxious attachment, avoidant attachment, frequency of parental physical victimization, witnessing mother hit father, emotion regulation difficulties, lack of emotional awareness, lack of emotional clarity, impulse control difficulties, difficulty engaging in goal-directed behavior, and nonacceptance of emotional responses.

Women who perpetrated physical IPV were more likely to have experienced parental violence victimization, witnessed both parents hit each other and their father hit their mother, reported more avoidant attachment, and more problems with impulsivity. Women who were victimized were also more likely to have experienced parental violence victimization, to have witnessed their mother hit their father, and to be both more anxiously and avoidantly attached and to be more impulsive.

Furthermore, women who reported higher levels of anxious and avoidant attachment and had more emotion regulation difficulties were more likely to perpetrate physical IPV than those who had anxious and avoidant attachment difficulties but had lower levels of emotional regulation difficulties. In addition, those women who reported higher levels of avoidant attachment were more likely to be victimized, and those reporting higher levels of anxious attachment were more likely to be victimized if they also reported high levels of emotion regulation difficulties and impulsivity.

These findings are consistent with previous reports with non-clinical samples that indicate most IPV occurrences are bidirectional [17,67] and that women who report unilateral victimization represent a lower percentage of women involved in violent relationships [21]. Findings are also consistent with studies showing that emotional regulation [43,51,52,53,54] and attachment difficulties are implicated in both perpetration and victimization of violence [38,39,40,68]. More specifically, the findings from this study support an association between avoidant attachment and perpetration and anxious attachment and victimization in young adult Hispanic females. Perpetration is also associated with attachment difficulties in women with significant emotional regulation and impulsivity difficulties. Both experiencing parental victimization and witnessing parental violence were associated with perpetrating violence and being victimized. The frequently obtained association between having experienced parental victimization and witnessed interparental violence and perpetrating violence against the partner as well as being victimized was also present in this sample of young adult Hispanic women. Interestingly, while witnessing parents hit each other and witnessing one′s father hit one′s mother was associated with perpetrating IPV, only witnessing one′s mother hit one′s father was associated with experiencing victimization of IPV. Further, witnessing father hit mother and mother hit father were highly correlated, suggesting that not only was the violence these young women reported in their relationships bidirectional but also that the violence they witnessed between their parents was bidirectional.

These findings also demonstrated that the physical IPV reported is mostly reciprocal, which has important implications for the treatment of IPV perpetrators. For instance, one such implication is that in cases where the violence is reciprocal, it may be an effective approach for professionals to involve both partners in an individual or conjoined intervention or treatment program. Both partners would benefit from learning important skills to address the identified risk factors in this approach. With young men and women in relationships characterized by physical aggression, several sessions of individual or conjoint treatment could involve insight into how one′s attachment style was learned, followed by sessions on how the attachment style can be changed and how to cope with the traits of an insecure attachment. For example, a brief motivational interviewing intervention has been used with college student couples to reduce partner aggression [69].

The need to address attachment issues, emotional regulation difficulties, experiencing parental violence, and witnessing interparental violence in childhood in young Hispanic women involved in violent relationships is further highlighted in these findings. As stated previously, relationship researchers view IPV as a dysfunctional attempt to remain close to a partner when threats to attachment needs are perceived [37]. Furthermore, perceived threats to attachment needs by insecure partners may generate differences in perspectives which can ultimately result in perpetration or victimization of violence or a combination of both. Therefore, it is important to develop interventions for both IPV perpetrators and victims that emphasize constructive ways to respond when these threats to attachment needs are activated. Furthermore, therapy could help them identify what their attachment needs are, how they could have been generated, how they influence their behavior in their relationships and how they interact with their partner′s attachment needs. These suggestions would be important to include as elements of interventions to help curtail the use of violence in relationships.

As stated previously, abundant evidence suggests that emotion dysregulation is a predictor of IPV perpetration, which may be due to not only a biological predisposition to react emotionally, but also to having been raised in an invalidating environment [44]. This study found that emotional regulation difficulties interact with attachment difficulties in violence perpetration and victimization within the relationship. It would be important for interventions to teach emotional regulation strategies to perpetrators and victims, particularly in situations where attachment needs are generated.

Lastly, consistent with previous research [55,56,57,61,64], witnessing interparental violence and experiencing childhood physical abuse were precursors of IPV perpetration and victimization in these young Hispanic women. These findings confirm that adverse childhood experiences are also a risk factor for IPV for young adult Hispanic women. These findings also support early prevention efforts targeting children who have either experienced parental violence or witnessed interparental violence and that address both emotional regulation and attachment issues in such interventions.

The findings of this study, largely confirm prior findings regarding IPV perpetration and victimization in non-Hispanic women. The percent of women in relationships characterized by aggression in this sample, 33%, is similar to other prevalence rates in college dating women [8]. Further, as noted in the introduction, variables found to be risk factors for IPV in this sample of Hispanic women are also risk factors of IPV in primarily non-Hispanic women samples such as emotional dysregulation [70] anxious attachment [32], and violence in one′s family of origin [71].

Although the present study added to the current literature on attachment, emotion dysregulation, exposure to violence in childhood and IPV among young adult Hispanic women, it is not without its limitations. The present study was conducted on a sample primarily recruited from a large Hispanic-serving institution in the Rio Grande Valley (RGV), a region in the U.S. state of Texas that is over 90% Hispanic and of Mexican American origin. This is both a strength and a limitation of the study. It is a strength because it is the first to study the risk factors and patterns of both IPV perpetration and victimization in young Hispanic adult women. However, these findings cannot be generalized to clinical or community Hispanic women in the RGV or other regions of the U.S. Additionally, the current study consisted of college students. Including young adult Hispanic women from the community would provide less selective findings and allow for greater generalization of the results. Additional limitations of the current research are its cross-sectional design, use of questionnaires (self-report data are subject to bias), and lack of romantic partner reports of the participants′ experience with violence in the relationship, either perpetrated or received. Future studies should address these limitations by replicating this research on predominantly Hispanic clinical or relationally distressed women and their partners.

## 5. Conclusions

This study reports on the frequency of IPV perpetration and victimization among young adult Hispanic women. The pattern of IPV for those women who report a history of violence in their relationships is one where the vast majority are involved in both perpetration and victimization of IPV. Indeed, the women who reported being only victimized represent a minute percentage of the sample. Women involved in bidirectional violence further report greater perpetration and victimization than the other women with a history of violence in their relationships. Having experienced violence from one′s parents, witnessing interparental violence, having an anxious or avoidant attachment, and having emotional regulation problems present risk factors for IPV perpetration and victimization in this sample of young adult Hispanic women. Findings support the need to include both dyad members in therapeutic attempts to address recidivism.

## Figures and Tables

**Table 1:** Sociodemographic profile of respondents.

Variables	Description	*n* (%)
Age (years) ^1^	18–24	308 (85.8)
	25–31	28 (7.8)
	32–38	12 (3.3)
	39–45	5 (1.4)
	45–51	6 (1.7)
Race/Ethnicity	Hispanic/Latino(a)	348 (96.7)
	Other	12 (3.3)
Relationship Status	Single	102 (28.3)
	Casually dating	34 (9.4)
	Seriously/exclusively dating	164 (45.6)
	Cohabiting	23 (6.4)
	Engaged	6 (1.7)
	Married	30 (8.3)
	Other	1 (0.3)
Cohabitation Status	Living with romantic partner	92 (25.6)
	Does not live with partner	268 (74.4)
Current/former Partner′s Sex ^2^	Male	339 (95.0)
	Female	17 (4.7)
	Other	1 (0.3)
IPV groups	Nonviolent	242 (67.2)
	Victim-only	15 (4.2)
	Perpetrator-only	39 (10.8)
	Bidirectionally violent	64 (17.8)

*Note*. Participants were on average 21.9 years old (*SD* = 5.39). ^1^ *n* = 359. ^2^ *n* = 357.

**Table 2:** Means and Standard Deviations of Scores on Risk Factor Measures by Total Sample and Physical IPV Groups.

	Total	Nonviolent	Victim ^1^	Perpetrator ^2^	Bidirectional ^3^
	*n* = 360	*n* = 242	*n* = 15	*n* = 39	*n* = 64
Attachment					
Anxious Avoidant	55.43 (22.92)	54.01 (23.35)	53.20 (20.89)	52.28 (19.97)	63.25 (22.19)
	49.04 (20.74)	47.11 (20.01)	48.47 (26.49)	44.82 (18.05)	59.05 (20.99)
Emotion regulation	86.79 (25.32)	84.88 (26.44)	94.60 (17.17)	84.21 (22.60)	93.85 (22.85)
Awareness	11.78 (4.56)	11.48 (4.55)	12.53 (3.81)	11.69 (4.78)	12.78 (4.55)
Clarity	86.67 (25.56)	84.88 (26.80)	93.87 (17.13)	82.72 (22.60)	93.95 (22.87)
Impulsivity	13.15 (5.10)	12.64 (5.01)	14.33 (3.92)	13.05 (4.64)	14.88 (5.61)
Goals	12.86 (5.49)	12.24 (5.38)	14.60 (4.21)	12.47 (4.99)	15.08 (5.93)
Non-acceptable	18.20 (6.87)	17.54 (7.05)	20.73 (5.51)	17.97 (6.73)	20.22 (6.11)
Strategies	3.71 (1.67)	3.61 (1.46)	3.47 (1.25)	3.68 (1.80)	4.17 (2.29)
PVV ^4^	2.33 (1.25)	2.19 (1.23)	2.40 (1.35)	2.15 (0.90)	2.95 (1.32)
WIV ^5^	0.58 (1.60)	0.44 (1.32)	0.47 (1.25)	0.60 (1.78)	1.11 (2.31)

^1^ Victim-only group. ^2^ Perpetrator-only group. ^3^ Bidirectionally violent group. ^4^ Parental physical victimization. ^5^ Witnessing interparental violence.

**Table 3:** Prevalence of Physical Violence Perpetration and Victimization by Category.

Physical Violence Reported		*n* (%)
No violence reported		242 (67.2)
Violence reported		118 (32.8)
	Bidirectionally violent	64 (54.2) ^1^
	Perpetrator-only	39 (33.1) ^1^
	Victim-only	15 (12.7) ^1^

*Note*. ^1^ Percentage is derived from participants that reported violence, *n* = 118.

**Table 4:** Correlations for IPV, Parental Physical Victimization, and Witnessing Interparental Violence.

Variables	1	2	3	4	5	6
Total IPV Perpetration (irrespective of victimization)	1	0.61 **	0.16 **	0.12 *	0.11 *	0.09
2. Total IPV Victimization (irrespective of perpetration)		1	0.22 **	0.09	0.10	0.12 *
3. Frequency of parental physical victimization			1	0.25 **	0.25 **	0.23 **
4. Witnessed both parents hit each other				1	0.90 **	0.84 **
5. Witnessed father hit mother					1	0.70 **
6. Witnessed mother hit father						1

* *p* < 0.05. ** *p* < 0.01.

## Data Availability

The data presented in this study are available on request from the corresponding author.
